# Choosing initial MS therapy; personal, disease, and medication factors^☆^

**DOI:** 10.1016/j.neurot.2025.e00582

**Published:** 2025-04-10

**Authors:** Bruce A. Cohen

**Affiliations:** Davee Department of Neurology, Northwestern University, Feinberg School of Medicine, Chicago, IL, USA

**Keywords:** Multiple sclerosis, Disease modifying therapy, Initial therapy

## Abstract

Initiating disease modifying therapy in a patient with newly diagnosed relapsing multiple sclerosis currently offers the best opportunity to influence their subsequent disease course. This article reviews personal factors, disease presentation characteristics, and data on current disease modifying therapies from the perspective of choosing initial treatment in this setting. Although metrics for prognostication at the individual level remain unreliable, particularly for those with mild presentations, currently available data on the relative efficacy of disease modifying therapies supports offering high efficacy therapy first line to most patients with newly diagnosed relapsing multiple sclerosis.

Multiple sclerosis (MS) is an inflammatory and degenerative disease of the central nervous system. It is considered the most common non-traumatic cause of neurologic disability in young and middle-aged adults [1,2]. It has also become one of the more successful stories in modern neurologic therapeutics, with the emergence of ten classes of medication, encompassing more than twenty individual agents, currently approved for treatment of relapsing forms of MS. Initiation of disease modifying treatment (DMT) of relapsing multiple sclerosis has been shown to modify the course of the disease, reducing the risk of disability progression for both relapsing onset [2,3], and more recently, progressive onset, multiple sclerosis [4,5]. While approved options for treating progressive onset MS are currently limited to a single agent, the choices are broader for the more common relapse onset presentations of MS. From the perspective of the newly diagnosed patient, and even for non-specialist treating clinicians, choosing among this multitude of options may appear overwhelming and complex [2]. This review will discuss factors to consider in guiding patients to successful initiation of a first disease modifying therapy, with the goal of optimizing their subsequent clinical course.

Before discussing issues related to medication choices, it is important to comment on the process by which both patient and clinician can successfully implement an initial course of treatment. Current therapeutic principles emphasize shared decision making for medical decisions, in which the patient's concerns and preferences are integrated with the physician's evidence-based recommendations, to collaborate on a treatment decision. Decisions should be patient centered and responsive to individuals' needs and values [6]. The American Academy of Neurology practice guidelines for initiating disease modifying treatment for multiple sclerosis recommend that the clinician counsel the patient regarding treatment options, and respect and incorporate patient preferences when initiating a DMT [7].

## Factors Contributing to the Initial Choice of DMT for MS

It is possible to organize the considerations impacting the choice of a specific DMT for MS into three overlapping domains; those related to personal preferences and characteristics, those related to the presenting features of the disease, and attributes of specific therapeutic agents.

### Personal factors

Patient values and preferences are heterogeneous with respect to concerns regarding potency, risks of adverse effects, impacts of different treatments on the quality of their life, and costs [8,9]. The diagnosis of MS presents a profound change to a previously healthy person. They now have concerns about the future course of an individually unpredictable illness, whether life and career goals must be altered, how a need for continuing treatment will affect their daily life, and financial considerations including the costs of the treatment and the potential financial impact of the diagnosis on insurance access and perhaps employment, now or in the future. They must now adapt to the identity of being a person with a chronic illness, while they still seek to maintain the normalcy of their life. How a person responds to the diagnosis, and need for initiation of treatment, including their perceptions of risks and benefits of medication, is shaped by the psychosocial context in which they live, including their trust in physicians, general coping responses and resilience to stress, support from family, friends, and faith organizations, and inclination to pursue independent investigation and information gathering [10]. Potential responses may range from denial of illness and offered treatment, to aggressive pursuit of medical therapy as a means of exerting control and providing hope for a better future course and a way to maintain normalcy [11].

The author finds it useful following delivery of the diagnosis to ask the patient what they know about MS, as a means of gathering insight into their initial reactions and potential fears. It may be helpful to probe for concerns regarding work or home life, and whether the patient has social support to help them adjust, by introducing a discussion advising initial discretion in disclosure of the diagnosis in work environments or other settings where the information might have unintended consequences.

Because making or confirming the diagnosis of MS is a stressful life event, it is wise to allow the patient time to process the diagnosis, provide an opportunity to discuss and affirm the emotions and fears that emerge, address individualized concerns, and importantly, convey reassurance that the availability of effective therapeutics offer hope in their potential to permit continuation and fulfillment of life plans. Because reaction to the imparting of the diagnosis may leave the individual less receptive to processing information at that time, it is best to leave the detailed discussion of specific treatment options to a follow up visit a short time later [7].

Education on the disease and treatment expectations and attributes are critical to enabling shared decision making and treatment adherence [12], and it is important to provide clear information on the disease, prognosis, and the role of DMT in maintaining normal life activities, in words easily understood by the patient. Education should begin when the diagnosis is made, and initially address both expressed concerns and the favorable changes in the natural history of MS when treated early with effective DMTs. It may helpful to note that while individuals with obvious impairments may come to the patient's mind, they are less likely to know about people with invisible symptoms of MS, who may be pursuing similar life and career aspirations without disclosing their diagnosis. It is also important to guide the person with newly diagnosed MS to reliable and unbiased sources of information, in anticipation of internet searches and seeking of opinions from others who may provide medical information based on limited knowledge and personal biases. Sources such as multiple sclerosis societies offer information on websites, as well as access to programs for newly diagnosed MS patients, which provide both disease information and support services. These sources also provide commercially neutral sources of information on available medications. The complexity of the current treatment environment can be overwhelming, yet research suggests that patients with MS desire more information about the disease and its management than they receive from their clinicians [6]. Individuals will vary in their weighting of risks and benefits as they consider medication choices [8,11,12], and the extent to which they prefer to engage in shared decision making [13]. The relative importance of perceived attributes of different medications may vary between patients and clinicians [14]. Routes and frequency of administration, impact of treatment on normalcy of daily life, infection risks, monitoring requirements, and potential side effects will vary in their impact on individual choices [8,9]. Age and gender may affect therapeutic choices [15]. Diverse beliefs about the medical system, and efficacy of treatment may reflect cultural and spiritual influences, and trust in the healthcare system and a clinician's medication recommendations [16,17].

An important consideration for women of childbearing age confronting a new diagnosis of MS, relates to plans for a future pregnancy, whether short term or at an undefined time in the future [8]. Counseling regarding possibility of a future pregnancy should always be included in the context of choosing an initial therapy. The newly diagnosed woman with MS should be educated that the disease and its treatment are compatible with future pregnancy, that pregnancy does not adversely affect the course of MS, and that when pregnancies are planned, outcomes are similar to those of comparable women without MS [18]. Women are generally advised to initiate DMT to establish disease control before pursuing pregnancy, and to use or continue effective contraception during that time period. Choices among initial disease modifying treatments will be influenced by their strategies for management, once pregnancy is to be pursued. Some agents will need to be discontinued prior to pursuit of pregnancy while others are safe to continue until pregnancy occurs. Some DMTs are compatible with post-partum nursing while others will need to be avoided during that period (reviewed in reference [18] and discussed below) Some highly effective therapies prior to pregnancy, and exclusive breast feeding, may influence post-partum relapse risk [19].

Finally, but importantly, it is an unfortunate reality that access to specific medications may vary based on an individual's health insurance coverage, or regulatory policies in some national health systems [20]. In some health settings, out of pocket costs can be a significant concern for patients with newly diagnosed MS and influence their treatment decisions [8,9]. Out of pocket medication cost may be defrayed by pharmaceutical support programs for some individuals, while others may be ineligible for support based on the source of their health care insurance. Formulary restrictions may limit access to preferred initial therapeutic choices For medications which are not self-administered, out of pocket cost for medication administration may be significant, even when the medication itself is provided without cost. Fortunately, a number of generic DMTs are now available at relatively low cost from large discount pharmacies and may offer treatment options to people otherwise unable to afford medication costs.

### Disease factors

Prognostication for future disability in patients presenting with newly diagnosed relapse onset MS remains challenging at the individual level [21]. Prognostic factors have been described in natural history and treatment cohorts, which are often used to inform DMT decision making in patients with relapse onset MS, however, the quality of evidence for published prognostic models is low [22].

Early natural history studies reported frequent relapses in the first two years of onset, and shorter intervals between early relapses, to be associated with shorter times to later disability [[23], [24], [25], [26], [27]]. A recent study also found that increased relapses, even if mild and non -disabling, were associated with increased risk of subsequent disability in untreated patients and those treated with lower efficacy DMTs [28].

More rapid accrual of disability as evidenced by presence of pyramidal signs, brainstem, cerebellar or cerebral deficits [23,25,27,29], incomplete remission from relapses (23.24.27), multifocal attack and symptom persistence [23,24,26,27,[29], [30], [31], [32]], increased age at onset greater than 35–45 [26,29], and cognitive impairment at onset [33], have also been associated with increased risk of later disability impairment. In an Italian cohort with pediatric onset MS, female sex and multifocal onset were risk factors for a second attack, and age younger than 15 and early DMT exposure decreased the risk of worsening disability. In comparison to an isolated brainstem or supratentorial syndrome, isolated spinal cord presentation or optic neuritis, or multifocal presentation was associated with a higher incidence of a first disability worsening event [34].

Literature suggests increased risk for disability in people of African ancestry [[35], [36], [37]], but reports are conflicting in Hispanic Americans [38,39]. Disparities in neurologic health care likely play a role in these outcomes [16,40,41].

Magnetic resonance imaging in patients presenting with a first clinical demyelinating event often reveals evidence of more extensive disease activity than apparent at clinical presentation. The extent of MRI involvement may have a stronger association with later disability than clinical features, particularly the presence of ten or more T2 lesions at presentation [42]. The presence of infratentorial brainstem and cerebellar lesions, and spinal cord lesions, have also been associated with increased risk of later disability in patients presenting with initial CNS demyelinating symptoms [43].

The classic fluid biomarker supporting the diagnosis of MS is the presence of unique oligoclonal bands in the cerebrospinal fluid. In addition to its value in supporting diagnosis, the presence of csf oligoclonal bands has also been associated with increased risk of later disability, compared to individuals without them at presentation [42].

Neurofilament light chains (NFLC) are markers of axonal damage which have been associated with both active inflammation, and risk of disability, in people with MS. Elevation of serum NFLC have been found in stored blood samples to precede initial symptoms of MS [44], and to increase prior to disability progression in patients with early MS [45,46]. NFLC are particularly sensitive to inflammatory disease activity and may have utility for monitoring efficacy of disease modifying therapy [47,48]. In patients with their first demyelinating event, high serum NFLC were associated with long term disability worsening [49]. Interpretation of NFLC results, however, may be confounded by similar elevations in neurodegenerative diseases, traumatic brain injury and aging [50], diabetes, impaired renal function, and tobacco smoking [51].

Elevations of glial fibrillary acidic protein (GFAP) levels are measures of astrocyte damage and possibly astrogliosis [52]. Increased GFAP levels have been found to associate with subsequent disability in both relapsing and progressive MS [53,54]. In contrast to NFLC, they are not thought sensitive to acute inflammatory activity [53,55]. A recent study found that elevated CSF, but not serum, GFAP levels obtained at onset of treatment in a cohort of patients with relapse and progressive onset MS was associated with increased risk for subsequent disability progression [56], hence the optimal fluid for assessing GFAP levels may not yet be certain.

Multicomponent serum assays for MS disease assessment are starting to emerge. By measuring multiple proteins with associations to MS disease processes, these assays may provide increased prognostic utility over single component metrics. One recent study demonstrated improved performance of an MS disease activity test panel, compared to NFLC, for prediction of new inflammatory disease activity, and may prove useful in estimating risk of future activity for treatment selection and monitoring [57]. Future panels might predict risk for disability progression to further inform early treatment decisions.

### Medication factors

Individual disease modifying medications for multiple sclerosis vary in efficacy when assessed in short term clinical trial cohorts. Earlier studies used comparisons to placebo in relapse onset MS, while more recent trials have employed active therapies as comparators. Cohorts in different trials perform differently, even when given similar therapeutic agents, for a variety of reasons, including changes in trial populations and MS diagnostic criteria, availability of established DMT options at the time of the study, unassessed and unrandomized comorbidity factors, and trial design differences [58]. To cite one recent example, the proportion of patients with three month confirmed disability progression events for teriflunomide 14 ​mg once daily in the original phase 3 randomized controlled trial (RCT) was 20.2 ​%, and the annualized relapse rate (ARR) was 0.37 [59], while for the same agent over a similar time period in the recent ublituximab trials in which teriflunomide was an active comparator, the three month confirmed disability progression rate was 5.9 ​%, and the ARR was 0.18 and 0.19 [60]. Nonetheless, available DMTs can be grouped into a hierarchy of therapeutic potency based on combined results of randomized trial and observational cohort studies [61]. Perhaps the closest approximation to the clinical setting of initiating a first DMT can be found in data from studies evaluating cohorts, or sub-cohorts, with a first clinical demyelinating event and naïve to prior therapy.

At the individual level, unknown genetic or biologic disease factors may affect individual response to a particular therapeutic class, and co-morbid conditions may affect the risk profile of particular agents. Monitoring requirements and concerns about adherence may favor one DMT choice over another for a particular individual. Even though general themes are emerging, the choice of a specific initial agent remains a decision best made in consideration of individual patient circumstances and clinician judgment.

The following paragraphs highlight aspects of each of the current U.S. FDA approved drug classes, from the perspective of their use as first line treatment choices for patients with newly diagnosed relapsing multiple sclerosis. At the time of this writing, only ocrelizumab is approved for treatment of primary progressive multiple sclerosis. These descriptions are not intended to be comprehensive presentations of the agents which can be found in other sources [62].

### Beta interferons

The approval of interferon beta-1b in 1993 marked the emergence of the DMT era for people with multiple sclerosis. Multiple formulations of interferon beta (IFNB) are currently available, all administered by injection either subcutaneously or intramuscularly. In randomized trials of patients presenting with their first clinical demyelinating event, the proportions of individuals initially treated with formulations of interferon beta who had a second clinical relapse at 2–3 years was 20.6 ​%–35 ​% [[63], [64], [65]]. In the interferon beta-1b study, 15 ​% of participants on active treatment from the onset of the study had EDSS progression within three years [66]. In a five-year extension study, the cumulative probability of a relapse in patients taking interferon beta 1a three times weekly was 39.2 ​% [67].

IFNB agents require injection administration and may cause mild or more serious injection site reactions. Initiation of treatment is usually accompanied by transient flu-like symptoms of myalgias, arthralgias, low grade fevers and fatigue. Patients should be monitored for hepatic enzyme elevations, and leukopenia, lymphocytopenia thrombocytopenia and thyroid dysfunction. The association of interferon therapy with depression is controversial, but reversible mood changes with interferon beta therapy may occur [68]. Interferon agents are now considered safe for use in pregnant women [18]. They are not immue suppressive and have not been associated with opportunistic infections such as progressive multifocal leukoencephalopathy (PML). Interferon beta agents have been in use for MS treatment for three decades without emergence of late safety concerns, but have diminished efficacy compared to newer agents as shown in direct clinical trial comparisons (see below).

### Glatiramer acetate

Glatiramer acetate (GA) was the second class of agents approved for treatment of multiple sclerosis. Administered by subcutaneous injection, GA showed similar benefits to interferon beta in randomized controlled trials, on relapse risk and time to first relapse [69,70], although the interferon group in one of the studies had fewer new MRI lesions [69]. In a study of patients with a first CNS demyelinating event, 24.75 ​% of subjects given GA developed a second clinical relapse during up to 36 months of follow up [71]. In a 12 month study of patients administering GA at 40 ​mg three times weekly, 23 ​% developed relapses [72].

Commonly reported side effects include injection site reactions and sporadic transient post injection reactions with symptoms of dyspnea, heat, tachycardia, chest discomfort and/or flushing, which are self-limited [72]. Lipoatrophy, resulting in permanent cosmetic changes, commonly occurs with prolonged use [73]. Anaphylactic reactions occur rarely but may be severe. GA is not immune suppressive and has not been associated with an increased risk of infections such as PML. Laboratory monitoring is not required, and the agent is safe for use in pregnancy [18]. Overall, GA offers a long-term safety record and limited monitoring requirements, but lesser efficacy than newer therapies.

### Teriflunomide

Teriflunomide is an oral DMT, approved in 2012, which has been shown to decrease relapse activity and reduce disability worsening in relapsing MS. Over 108 weeks, annualized relapse rate (ARR) for the 14 ​mg oral daily dose was 0.37 with relative risk reduction of 31.5 ​% versus placebo. The proportion of patients with 12 week confirmed disability worsening was 20.2 ​% for this dose. [59]. As illustrated above, relapse rates, and rates of disability worsening have been lower in more recent studies in which teriflunomide was used as an active comparator, although cohorts recruited in different treatment eras may be subject to selection bias for individuals with less active disease. In a study of patients with a first demyelinating event, the cumulative rate of relapse at 108 weeks for teriflunomide 14 ​mg orally once daily was 24 ​% [74]. In a small cohort of patients with radiologically isolated syndrome, teriflunomide was recently shown to reduce the risk of a clinical event compared to placebo by 72% over a 3-year period [75].

Teriflunomide is generally well tolerated but requires initial monthly hepatic enzyme monitoring for the first 6 months, commonly causes transient hair thinning during the first 6 months, and can cause headache and gastrointestinal side effects. Less commonly, it can cause hypertension and peripheral neuropathy with paresthesias. A major consideration regarding its use as an initial therapy is its teratogenic potential, which requires effective contraception in both women of childbearing potential and men who have partners who may become pregnant. Additionally, due to enterohepatic recirculation, it is eliminated very slowly and must be extracted with cholestyramine in the event of a need to rapidly discontinue its use [76].

Teriflunomide offers once a day oral dosing and long-term safety and tolerability [76,77]. It is considered a lower efficacy therapy and has been used as a comparator agent in recent clinical trials (see below). It is now available in the US as a generic formulation at greatly reduced cost from discount pharmacies.

### Fumarates

Dimethyl fumarate (DMF) is an oral DMT approved for relapsing MS in 2013. Taken twice daily (bid), it showed benefit, compared to placebo, in randomized clinical trials, in reducing relapse rate, proportion relapsing and proportion with 3 month confirmed disability, over a two-year period. Annualized relapse rate for the bid dose group was 0.17, and proportion with confirmed progression of disability was 16 ​% [78]. In a second parallel study, ARR over 2 years was 0.22. In this study, a reference arm compared GA to placebo and found a lower reduction of ARR compared to placebo for DMF than for GA, however, the study was not powered to compare the two active treatment arms [79]. In a post hoc analysis, for individuals with newly diagnosed multiple sclerosis within one year and naïve to DMT, ARR at 2 years was 0.17 and estimated proportion with 12-week sustained disability progression was 0.07 for the twice daily dosing group [80]. In a small cohort of people with radiologically isolated syndrome, dimethyl fumarate reduced the risk of a clinical demyelinating event over 96 weeks compared to placebo by 82%, with three events in 44 patients [81]. In a 96-week randomized trial, compared with IFNB-1a in pediatric onset MS, DMF was superior in prevention of new/enlarging T2 lesions and in reducing ARR which was 0.24 in the DMF group [82].

Common side effects with DMF are flushing, and initial gastrointestinal symptoms which tend to resolve with continued use, and lymphopenia in about 10 ​% of patients. Serious infections are rare but can occur irrespective of the leukocyte count [83]. Progressive multifocal leukoencephalopathy has been rarely reported in dimethyl fumarate treated patients [84]. Formulations of monomethyl fumarate, the active metabolite of DMF, and diroximel, which is converted into monomethyl fumarate, also taken twice daily are available and may be associated with less initial gastrointestinal symptoms.

Advantages of this class of agents include a long-term and relatively benign safety profile, oral administration, short half-life allowing rapid discontinuation, and moderate efficacy. Now that generic DMF is available, cost is low when purchased directly from discount pharmacies. Disadvantages include less potency when compared to newer agents, and the need for twice daily dosing. Fumarates are generally categorized as moderate efficacy agents and have been compared indirectly to higher efficacy DMTs in recent observational studies assessing comparative outcomes of initial treatment strategies (see below).

### Sphingosine 1 phosphate modulators

Fingolimod, the initial drug in this class, was the first oral agent approved for treatment of relapsing multiple sclerosis in 2010. Randomized clinical trials in relapsing MS patients showed reductions in annualized relapse rate (0.18 vs 0.40) and probability of disability progression confirmed at three months (17.7 ​% vs 24.1 ​%) for the 0.5 ​mg approved dose compared to placebo [85]. In addition to reduction of new inflammatory lesions on MRI, fingolimod also was effective in decreasing the rate of brain volume loss [85]. In a study comparing fingolimod to once weekly interferon beta-1a, fingolimod was more effective in reducing annualized relapse rate over 12 months which was 0.16 for fingolimod 0.5 ​mg compared to 0.33 for interferon beta-1a [86]. In a study of pediatric patients with relapsing MS, fingolimod was superior to weekly intramuscular interferon in reducing relapse rate. In a study comparing fingolimod to GA, fingolimod 0.5 ​mg daily was more effective in reducing annualized relapse rate which was 0.15 compared to 0.26 for GA over 12 months [87].

Fingolimod is believed to work as an anti-migratory agent by restricting access of lymphocytes activated in lymph nodes to the circulation through binding and internalization of the S1P-1 receptor, thereby preventing their access to the CNS [88,89]. Pre-clinical data supports an effect on glial cells which express S1P-5 receptors, although this has not been shown in humans [90]. A study of fingolimod in primary progressive multiple sclerosis failed to show benefit over placebo in preventing disability progression [91].

Fingolimod is contraindicated when pursuing pregnancy. [18]. Rebound relapses can occur following discontinuation and may be more likely in younger patients with lower lymphocyte counts and longer exposure to the medication [92,93]. Risks of infection are increased in people taking this medication [94,95], with some potential for serious infections, notably including PML [96], and cryptococcal infections [97]. Response to vaccines is diminished but not eliminated, and live vaccines are contraindicated, when taking this medication and until repletion of lymphocytes following discontinuation [98].

Because the S1P receptor is widely expressed among its five subtypes, off target effects occur with fingolimod. Transient first dose effects on the cardiovascular system, including hypotension, bradycardia, transient conduction block and arrhythmias, require first dose monitoring. Rarely the agent can cause macular edema, and pretreatment screening, and post treatment ophthalmologic evaluation are required. The risk of macular edema is increased in people with diabetes and patients with a history of uveitis, requiring regular ophthalmologic monitoring [99]. An increased risk of cutaneous malignancies is also associated with fingolimod, and regular dermatologic screening is recommended. Additional adverse effects include hepatotoxicity and hypertension [89,100].

Additional agents in this class have been developed with more selective targeting of sphingosine receptor subtypes and have employed initial titration regimens to eliminate the need for first dose observations in patients without cardiovascular disease. Risks of macular edema are reduced but not eliminated with these agents. Other adverse effects overlap with fingolimod, with some additional considerations noted below.

Siponimod and ozanimod selectively target S1P-1 and S1P-5 receptors. Siponimod was evaluated in patients with secondary progressive MS. Compared to placebo, Siponimod reduced the risk of confirmed 3-month disability progression by 21 ​%, the primary outcome, and 6 month confirmed disability progression by 26 ​% [101]. In an open label extension, six month confirmed disability progression risk was reduced by 22 ​% in continuously treated patients, compared to those initially randomized to placebo, and risk reduction was greater in those with relapses or MRI activity prior to entry into the study [102]. A reduction of 23 ​% was found on the risk of worsening cognitive processing speed for the continuously treated group [102]. Siponimod requires CYP2C9 genotyping prior to dosing and is contraindicated in those homozygous for the type 3 genotype. Reduced dosing is required for individuals heterozygous for this genotype. Other medications inducing or inhibiting this enzyme can result in drug interactions, including modafinil, which is not recommended for concomitant use. Siponimod has a short half-life of approximately 30 ​hours and is rapidly eliminated following discontinuation. It is indicated for relapsing MS and active forms of secondary progressive MS [100].

Ozanimod was compared to weekly IFNB-1a in relapsing MS and proved superior in reducing relapse rates and new inflammatory MRI lesions in two clinical trials. Annualized relapse rates for ozanimod 1.0 ​mg orally once daily were 0.17, compared to 0.28 for interferon beta over 24 months [103], and 0.18 for ozanimod 1.0 ​mg versus 0.35 for interferon beta over 12 months [104]. No difference in disability progression was found in these studies. In an extension study, 12 ​% had a serious treatment emergent adverse event (TEAE), 2.8 ​% had serious infections and 2.8 ​% had cardiac events. Over 48 months, 71 ​% remained relapse free and frequency of new MRI lesions remained low [105]. Although ozanimod has a short half-life, it has a longer acting active metabolite with a half-life of 10 days. Concomitant use of opioids, selective serotonin reuptake inhibitors, selective norepinephrine reuptake inhibitors, and tricyclic agents are not recommended, although a post hoc analysis of the DAYBREAK extension study did not find an increased number of serotonin syndrome related adverse events [105].

Ponesimod selectively targets S1P-1 and was superior to teriflunomide 14 ​mg daily in reducing relapses and inflammatory MRI activity, but not risk of disability progression, over 108 weeks. Annualized relapse rates for ponesimod 20 ​mg daily were 0.202 compared to 0.290 for teriflunomide [106]. Ponesimod has a short half-life and is eliminated within two weeks [100].

Fingolimod is variably considered to be either moderate or high efficacy by different authors [61]. It offers advantages of once daily oral dosing but requires significant safety monitoring and has potential risk for serious infections. When discontinued, it can be associated with rebound relapse activity, which can be an issue when discontinuation may be anticipated for future pregnancy. Newer agents in this class target fewer receptor subtypes and may have less off-target side effects offering advantages over fingolimod. They do not require first dose monitoring due to titration schedules, and those with shorter half-lives do not appear to be associated with rebound on discontinuation. This class of agents offers an oral therapy with relatively high efficacy, but may not offer advantages compared to some alternate high efficacy DMTs with less extensive adverse effect profiles and equivalent or superior efficacy. Comorbid conditions may impact suitability of S1P agents for use in particular individuals, and potential interactions with commonly used medications in MS patients occur with some agents in this class. Fingolimod is now available as a generic medication in the US.

### Cladribine

Cladribine is an oral cytolytic agent, which is metabolized and taken up by lymphocytes resulting in selective accumulation of 2-chlorodeoxyadenosine-5' -diphosphate (2-CdATP) due to lack of 5′ nucleotidases in these cells. Incorporation of 2-CdATP into cellular DNA interferes with DNA synthesis and repair leading to cell death [107]. Cladribine is taken orally in two courses a month apart dosed according to weight. After 48 weeks, the dosing cycle is repeated. In a randomized clinical trial, cladribine reduced annualized relapse rates to 0.14 for the approved 3.5 ​mg/kg dose arm compared to 0.33 in placebo treated individuals, reduced new MRI lesion activity, and reduced risk of three month confirmed disability progression by 33 ​% [108]. In an extension trial in participants who were given no additional active therapy, 75 ​% remained relapse free over 2 years [109]. A separate study in patients with a first clinical demyelinating event was terminated by the sponsor before completion, however, it did show a reduction in the number of individuals with subsequent relapse of 13 ​% in the 3.5 ​mg/kg treatment group compared to 34 ​% in the placebo group [110]. In a retrospective observational study of a prospective cohort, cladribine treatment was associated with a lower annualized relapse rate than fingolimod, but a higher rate than natalizumab, alemtuzumab, or ocrelizumab. Risk of disability worsening for cladribine did not differ from fingolimod or alemtuzumab but was higher than natalizumab and ocrelizumab [111].

Potential adverse effects of cladribine include immune suppression, infections, hepatotoxicity, persistent lymphopenia and teratogenicity. Transfusion associated graft versus host disease has rarely been observed in MS patients who have been treated with cladribine and it is recommended that only irradiated blood be administered to individuals who have been treated with this agent. Cladribine carries a black box warning for malignancy and teratogenicity, and the US FDA considers it generally recommended for patients who have had an inadequate response to, or are unable to tolerate, an alternate drug indicated for the treatment of MS. It is not recommended for use in clinically isolated syndrome because of its safety profile [112].

### Natalizumab

Natalizumab was the first monoclonal antibody approved for treatment of multiple sclerosis. Compared to placebo, it was effective in reducing relapses by 68 ​%, new T2 activity on MRI by 83 ​%, and the risk of disability worsening by 42 ​% over two years [113]. Initially given intravenously every four weeks, a recent study found extended interval dosing at 6 weeks similarly effective in most patients [114]. Natalizumab is an anti-migratory agent which targets alpha 4-beta1- integrin expressed by activated lymphocytes to bind vascular cell adhesion molecules at the endothelial cell membrane, allowing them to adhere and then migrate into the CNS. CSF studies in patients taking natalizumab show diminished numbers of T and B lymphocytes [115]. The major adverse effect of this strategy is a risk of CNS infections, particularly PML. The risk of PML is increased in patients taking natalizumab who were previously treated with immune suppressive therapies, or after longer durations of treatment [116]. A reliable biomarker for PML risk in patients treated with natalizumab, the anti JCV antibody index, is now available and can be used to predict risk of PML at the individual level in patients not previously treated with immune suppressive therapy [117,118]. Individuals seronegative for this antibody have extremely low risk of PML, however, seroconversion occurs, and the antibody must be re-tested at least every 6 months during therapy. Periodic brain MRI is also recommended in patients taking natalizumab as discovery of PML prior to symptom onset is associated with improved prognosis [119]. Other side effects include infusion reactions which may be related to anti-natalizumab antibodies, and rare hepatotoxicity [115]. Cases of other CNS infections have been reported in patients taking natalizumab.

Natalizumab effects on peripheral immune cells reverse within 16 weeks following discontinuation [120]. Discontinuation of natalizumab is associated with potential rebound attacks which can be severe and prevention requires overlap of an alternate DMT with a rapid onset of action [121].

Natalizumab is not approved for use in pregnancy, although some studies suggest it may be safe during the first and second trimesters. Later in the pregnancy, natalizumab administration may be associated with anemia and thrombocytopenia in the neonate. Use while nursing is not approved, but literature suggests that infant exposure through nursing is low, due to limited transmission to breast milk and breakup of any ingested antibody in the stomach [122].

Natalizumab offers a highly effective therapy for relapse suppression and reduction of future disability risk, with a reasonable safety profile in anti JCV antibody seronegative individuals. Natalizumab has high monitoring requirements and requires relatively frequent infusion dosing. Anti -JCV antibody status must be monitored because seroconversion occurs and may advise discontinuation of the drug. Because of potential rebound disease activity, gaps in therapy and discontinuation may put patients at risk, unless an alternate DMT with a rapid onset of action is started to provide overlapping protection. In contrast to cytolytic agents, natalizumab is not a systemic immune suppressant, and immune response to systemic infections and vaccines are not impaired.

### Alemtuzumab

Alemtuzumab is a potent monoclonal antibody targeting CD52 which is present on both T and B lymphocytes. Although the U.S. FDA does not recommend it for CIS and considers it generally indicated in individuals who have failed to respond to other therapies, it has been used as first line therapy in the United Kingdom. One of the pivotal phase 3 trials for alemtuzumab enrolled patients with active MS, naïve to prior treatment, and within five years of onset (CARE-MS-1). This study used interferon beta-1a as an active comparator, and hence, represents the first randomized controlled study comparing a high efficacy therapy to a lower efficacy agent as first line treatment. Administered by infusion in two annual cycles, alemtuzumab results in rapid lysis of blood lymphocytes, associated with significant infusion reactions requiring pre-infusion prophylaxis. In CARE-MS-1, alemtuzumab was found superior to interferon beta-1a three times weekly in reducing relapse rate by 55 ​% over 2 years, with 78 ​% of patients relapse free [123]. In an extension study over 5 years which allowed retreatment for emergent disease activity, 68.5 ​% received no additional therapy. At five years, 60 ​% had stable disability scores, 18 ​% were worse, and 22 ​% were improved compared to baseline [124]. In an open label follow on study entered by 68 ​% of the original participants, of those receiving alemtuzumab in the randomized study, 65 ​% were free of 6 month confirmed disability progression, and 42 ​% showed 6 month confirmed disability improvement, over a period of up to 13 years following initial treatment, which did not differ significantly from those assigned to IFNB during the RCT and switched to alemtuzumab on entry into the follow on study. The ARR for years 3-13 was 0.14 in both groups. Freedom from MRI activity during years 3–11 was observed for 28 ​% of those originally randomized to alemtuzumab [125].

Alemtuzumab has a significant adverse event profile. Severe infusion reactions can occur, and treatment requires prophylaxis with steroids and antihistamines prior to infusion. Secondary autoimmune diseases appear in more than 40 ​% of patients, most commonly Grave's disease, but also immune thrombocytopenia, and glomerulonephropathy due to anti-glomerular basement membrane antibodies. Infectious complications include herpes simplex infections during the initial months following infusion for which prophylaxis is recommended [126], and listeria infections for which dietary restrictions have been recommended for patients taking this medication [127]. Additional reported adverse effects include hemolytic anemia, rare cases of cerebral artery dissection and stroke in the peri-infusion period, thrombotic microangiopathy, acute coronary syndrome, acalculous cholecystitis, and a case of PML [128]. The US FDA lists warnings for secondary autoimmune conditions, serious and life-threatening infections, stroke, and possible increased risk of malignancies, as black box risks [129]. A risk management program requires monthly blood and urine screening for four years following the last dose of alemtuzumab.

Alemtuzumab is a highly potent DMT with prolonged suppression of inflammatory MS activity in many patients, however, breakthrough disease occurs in a substantial number of individuals following initial therapy, requiring either retreatment with the agent or an alternate DMT, and serious adverse effects may occur with its’ use. Intensive monitoring is required for years following the last administration of the agent, which can be difficult to implement when patients move or otherwise lose contact with the administering center. The emergence of newer treatments has provided safer and more easily monitored alternatives for high efficacy therapeutic strategies.

### B-cell depleting therapies

B cell depleting agents for MS act on lymphocytes in blood, predominantly B lineage cells, which carry the CD20 epitope. These monoclonal antibodies bind the cell and induce destruction through antibody mediated cytotoxicity, or complement dependent cytotoxicity, inducing a selective immune suppression. Re-emergence of lymphocytes into the blood requires periodic re-infusion or injection to maintain benefit [130]. Analysis of pathological material has shown follicle-like aggregates in the meninges of people with secondary progressive MS, associated with underlying cortical pathology, implicating B- lymphocytes in mechanisms of progressive worsening in the disease [131].

Ocrelizumab was the first of these agents to be approved for use in MS. Administered intravenously at six-month intervals in two parallel clinical trials, ocrelizumab was 46/47 ​% more effective than interferon beta-1a subcutaneously three times weekly, in reducing relapses (ARR 0.16 vs 0.29), and in a pooled analysis, 40 ​% more effective in reducing confirmed disability worsening at 24 weeks (6.9 ​% vs 10.5 ​%) in people with relapsing MS [132]. A separate study in patients with progressive onset MS showed a 24 ​% reduction in risk of disability progression at 12 weeks (32.9 ​% vs 39.3 ​%) and 25 ​% reduction at 24 weeks (29.6 ​% vs 35.7 ​%) compared to placebo [133]. Benefits in both populations have been maintained in extension studies in which patients receiving ocrelizumab in the randomized portion of the trials have done better than the comparator groups switching to ocrelizumab treatment for the extension [134,135]. In a subgroup of participants receiving ocrelizumab as their first therapy for relapsing MS, 48 ​% had no evidence of disease activity clinically or by MRI, and 79 ​% had no evidence of 24 week disability progression over nine years of follow up [136].

As this is being written, a subcutaneous formulation of ocrelizumab has been approved by the U.S. FDA for use in patients with either relapse onset or progressive onset MS. This formulation is also dosed every six months and should be prepared by a health care professional and then injected over 10 ​min immediately following preparation [137]. In a phase 3 randomized, open-label, controlled, study, 920 ​mg subcutaneous administered ocrelizumab demonstrated non-inferiority compared to intravenous 600 ​mg ocrelizumab on clinical relapse and new MRI activity outcomes [138]. The most common adverse events with subcutaneous ocrelizumab were injection reactions with 47 ​% experiencing local injection site reactions and 11 ​% systemic reactions with the first dose. Most occurred within 24 ​h and all were mild or moderate in severity. Treatment was required in 26 ​% and median duration was three to three and a half days. Subsequent doses were associated with injection site reactions in 31–43 ​% and systemic reactions in 3–7 ​% over the second through fourth injections [137].

Ofatumumab is a more humanized anti-CD20 monoclonal antibody, given by self-administered subcutaneous injection every 28 days. In a phase 3 randomized clinical trial, it reduced annualized relapse rates by 50 ​% (0.11 vs 0.22, and 0.12 vs 0.25) compared to teriflunomide 14 ​mg daily. Ofatumumab reduced the risk of 3- and 6-month disability worsening by 34 ​% (10.9% vs 15% ) and 32 ​% (8.1% vs 12%) respectively [139]. In an open label extension study, recently diagnosed, treatment naïve, individuals receiving ofatumumab beginning with assignment in the randomized study, had annualized relapse rates of 0.1-0.01 over five years [140].

Ublituximab, is glyco-engineered to decrease fucose content and increase FC¥RIIIa receptor binding and activation of natural killer cells. In paired phase 3 trials in relapsing MS, ublituximab reduced adjusted annualized relapse rates over 96 weeks by 59 ​% (0.08 vs 0.19) and 49 ​% (0.09 vs 0.18) compared to teriflunomide 14 ​mg daily, with parallel benefits on new MRI activity, but was not more effective in reducing the risk of disability worsening which was low in both groups (5.2 ​% vs 5.9 ​%) [60]. In a subset of treatment naïve participants within three years of their first symptoms of MS, improvements in expanded disability status score and multiple sclerosis functional composite score from baseline were greater in those receiving ublituximab, compared to those starting treatment with teriflunomide [141].

Rituximab, which is considered to work in a similar fashion to ocrelizumab, is used in many parts of the world, and in some US centers, although not approved by the US FDA for use in MS. A recent observational study compared rituximab 500 ​mg every six months to other DMTs on clinical disability progression, MS related quality of life, and clinical relapses and new MRI activity. Results showed benefits for rituximab over injectable and oral comparators, but not over natalizumab, for outcomes other than clinical disability worsening, which was low [142]. Another study showed superior clinical efficacy for rituximab over injectable DMTs and dimethyl fumarate as initial therapy for relapsing MS [143]. A recent observational cohort study failed to show non-inferiority of rituximab compared to ocrelizumab for relapse prevention, however, no difference in disability worsening was seen between the arms [144].

The most common side effects of the b cell depleting agents are infusion reactions for the intravenous formulations, and cutaneous reactions for the subcutaneous administered agents. Infection risk is increased, particularly for respiratory tract infections. Most, but not all, infections are mild, and rates are similar to those in active control agents [134]. Lower respiratory tract infections and other more serious infections may occur in some individuals. Latent Hepatitis B infection may reactivate in patients on b-cell depletion therapies and screening is required prior to use. Serious opportunistic infections such as PML have been rare. Most PML cases have occurred in patients transitioning from natalizumab or fingolimod to ocrelizumab, and still in the risk window for the prior drug [145]. Long term use of ocrelizumab and rituximab have been associated with the emergence of hypogammaglobulinemia, which if severe, can further increase risk of infection [146]. Recently, cases of immune mediated colitis have been described in patients taking ocrelizumab [147].

B cell depleting agents attenuate response to vaccines, however, a response is preserved from persisting T lymphocytes. Live virus immunizations cannot be given to patients taking these medications [148]. A concern for potential increased risk of malignancies, particularly breast cancer, emerged in the initial clinical trials. Longer term follow-up of the cohorts has suggested that this risk is not different from the background population risk [149], although the U.S.FDA has not removed the caution regarding possible increased risk of malignancy at this time.

Although not approved by the FDA for use in women pursuing pregnancy or nursing infants, the intravenous formulation of ocrelizumab offers prolonged biologic effects beyond its pharmacologic clearance, which can accommodate planned pregnancy timing [18]. Discontinuation of ocrelizumab is not associated with rebound MS activity. Ocrelizumab and rituximab have been shown to have little penetration into breast milk, and small risk of impact on nursing infants, potentially allowing resumption of treatment two weeks following completion of the pregnancy [150].

The B cell depleting agents offer high efficacy treatment options with reasonable risk profiles and monitoring schedules, compared to other highly effective DMTs, for patients with relapse onset MS. The administration schedule and prolonged efficacy of the intravenous formulations are compatible with planned pregnancies, an important consideration for many women at the common ages of onset for relapsing MS. Because meningeal lymphocyte aggregates are a feature of progressive MS, these therapies may impact pathogenic mechanisms of progression independent of relapses. Benefits of ocrelizumab for progressive onset MS are modest but, at this time, represent the only DMT with demonstrated benefit on disability worsening in an RCT approved for use in patients with this presentation.

## Therapeutic strategies for initial MS DMT

Therapeutic strategies for initial MS treatment include escalation, or initial high efficacy therapy. Escalation strategies initiate a moderately potent agent with a modest side effect profile based on the premise that some individuals will respond well to the first therapy, as seen in earlier treatment eras when more limited options were available, and that the risk of serious and/or long term side effects will be less [2]. In addition to safety monitoring, the strategy requires monitoring for efficacy by clinical and MRI imaging. Ideally a target for efficacy is set and if the patient's disease activity exceeds the target, treatment is switched to a higher efficacy agent. As more effective DMTs have emerged, the target has shifted towards minimal or no evident disease activity (MEDA/NEDA) consisting of the absence of clinical relapses, minimal or no new lesions on MRI, and no changes in disability based on examination and functional performance measures [61,151]. The strategy requires that both patient and clinician are attuned to potential symptoms of disease activity and the need for timely evaluation to confirm emergent clinical symptoms represent instances of true breakthrough on current therapy and not pseudo-relapses. An important limitation to this strategy in the current era is the inability to predict MS disease course at the individual level and the possibility that a breakthrough relapse will result in residual impairment [151].

Initial treatment with high potency therapy offers the opportunity to maximize suppression of MS related inflammation as early in the course of disease as possible, when the impact of relapse reduction has been shown to offer the most benefit on disability progression [[151], [152], [153]], in a population less likely to have co-morbidities increasing the risk of therapy, and having the potential for initial high efficacy therapy to confer lasting benefits on mechanisms of subsequent disability progression. A consideration with this strategy is the potential risk for serious adverse effects and limited data on long term safety of high efficacy therapies which must be weighed against the reduced risk of disability progression for the specific agents of interest [61].

Some observational data has emerged comparing disability outcomes between patient cohorts initiated on high efficacy therapies to groups initiating moderate efficacy agents. A study using the MSBase and Swedish MS registries, compared individuals starting high efficacy infusion therapies within two years of disease onset to those starting these therapies later, on subsequent disability at 6–10 years, using propensity score matching. The authors report a benefit on subsequent disability favoring the early high efficacy treatment group persisting each year through the tenth year of treatment [154]. In a propensity matched cohort from a Danish registry, initial high efficacy therapy which included fingolimod, was associated with 47 ​% reduced rate of disability worsening, and a 50 ​% reduced rate of first on treatment relapse, compared to the moderate efficacy initial treatment over 4 years of follow up [155]. In a propensity matched cohort of 1825 Finnish patients, initial treatment with high efficacy infusion therapy, compared to moderate efficacy agents, reduced the risk of disability progression confirmed at 6 months by 40 ​%, and first on treatment relapse by 30 ​% over 5 years of follow up [156]. An observational cohort study found a lower risk of conversion to secondary progressive MS in patients initially treated with fingolimod, natalizumab or alemtuzumab, compared to those starting treatment with interferon beta or glatiramer acetate, with a hazard ratio of 0.66 ​at 5 years [157]. In open label extension studies of patients receiving ocrelizumab for relapsing MS, 76.6 ​% were free of 48 week confirmed disability progression after 10 years of follow up. The risk was lower in those starting ocrelizumab compared to those switching to ocrelizumab for the open label extension (HR ​= ​0.77) [149].

Two currently ongoing randomized controlled studies are addressing the question of whether high efficacy initial therapy is more effective in all patients, regardless of presenting risk factors; Traditional versus early aggressive therapy for multiple sclerosis trial- TREAT-MS, NCT 03500328; and Determining the effectiveness of early intensive versus escalation approaches for RRMS, DELIVER MS NCT 03535298. The TREAT-MS study is stratifying enrollment by baseline risk factors. Until data from these ongoing studies is available, and validated biomarkers to inform individual level prognostication emerge, available data from direct treatment comparisons, and consistent findings in observational cohorts discussed above, support initiation with a higher efficacy therapy in most patients with newly diagnosed relapsing multiple sclerosis and should be considered and offered in the absence of contraindications.

### Selecting a first DMT for the individual; the Author's approach

The author prefers to defer discussion of initial treatment choices to a separate visit shortly following the initial evaluation and delivery of the diagnosis of MS. At the time of the initial visit, education on the disease and benefits of DMT is introduced, and screening laboratory studies for potential treatment options are obtained. Prior to the second visit, a short list of three or four agents are selected for presentation based on co-morbidities, potential pregnancy considerations, patient expressed preferences, any known access limitations and results of the screening labs. Typically, choices will include both high efficacy and at least one moderate efficacy agent.

Most patients will not be interested in agents requiring frequent injections, except for those individuals who are so risk averse, that they will prefer glatiramer acetate. However, as this is being written, the U.S.FDA has issued a black box safety warning regarding rare cases of serious anaphylactic reactions in patients using GA, even after prolonged exposure [158], which will now need to be discussed when presenting this option.

I do not use teriflunomide in women of childbearing age or their partners, due to its potential teratogenicity, however, it is a reasonable option for patients with later onset mild MS who favor a low-risk option.

I do not offer S1P modulators to patients with comorbid cardiovascular disease, uveitis, or diabetes mellitus, but will consider more selective options in this class for someone who wants a once daily oral agent. In women of childbearing age, I would consider siponimod or ponesimod because of their shorter half-lives. Ozanimod is an attractive option for a patient with both MS and inflammatory bowel disease as it is approved for ulcerative colitis and has shown benefit in studies of Crohn's disease.

I offer a B-cell depleting agent to any patient without a contraindication, although I prefer other options if screening labs show baseline hypogammaglobulinemia or Hepatitis B virus infection. In patients with latent Hepatitis C virus infection, treatment of the hepatitis can be accomplished in a few months allowing a strategy of initiating a short term bridging agent with a planned switch to a B-cell depletion therapy following successful clearance of the HCV.

I will offer natalizumab as an alternate high efficacy therapy in patients who have a negative anti-JCV antibody index, and some may prefer this option over a broader, albeit selective, immune suppressive therapy.

I do not consider alemtuzumab for first line treatment of MS, even in patients with active presentations, preferring natalizumab or a B-cell depleting agent in that setting. I also generally avoid cladribine as first line therapy, unless other high efficacy treatment options are limited by individual factors, although it can be considered in settings where frequent therapeutic monitoring is challenging.

I initiate the treatment discussion by presenting goals and expectations for DMTs and explain and review comparative data on escalation and high efficacy initial therapeutic strategies, noting that these represent group comparisons and that individuals may differ in their response to any specific agent. I present the selected options in each category reviewing their efficacy, monitoring requirements, and potential adverse effects. After addressing questions, I present my recommendation to the patient, usually a high efficacy therapy. I provide time for them to consider the options and do their research if desired, directing them to sources such as the website for the National Multiple Sclerosis Society for commercially neutral information. We agree to talk again within a week to initiate treatment.

## Concluding Comments

Early initiation of effective disease modifying therapy in a patient with new onset relapsing multiple sclerosis currently offers the best opportunity to influence the subsequent disease course. The implementation of initial treatment takes place during a period of disruption in the patient's life, with personal factors impacting their perceptions, and influencing decisions regarding treatment for the disease. It is important to provide education and a reference point for reliable information on the disease, its prognosis, and particularly on the impact of current DMTs on the natural history of the disease. While disease factors have been identified, which are associated with increased risk of clinical worsening, the prognosis for individuals with mild presentations is presently difficult to assess accurately. Randomized studies are underway to evaluate the relative benefits of high vs moderate efficacy initial therapy for MS, however long term follow up will be needed to adequately inform sustained benefits and any long term emergent risks. At the present time, comparative trial data and observational data support offering high efficacy therapy to most patients with newly diagnosed relapsing MS, in the absence of contraindications.

## Author contributions

Author is 100% responsible for contents.

## Declaration of competing interest

The authors declare that they have no known competing financial interests or personal relationships that could have appeared to influence the work reported in this paper.
